# Kinematic and Physiological Analysis of Medieval Combat Sport Using Motion Analysis, Blood Lactate Measurement, and Heart Rate Monitoring: A Case Study

**DOI:** 10.3390/s24113443

**Published:** 2024-05-27

**Authors:** Mojtaba Mohammadalizadeh, Suraj Jaiswal, Scott Semken, Aki Mikkola

**Affiliations:** Laboratory of Machine Design, Department of Mechanical Engineering, LUT University, 53850 Lappeenranta, Finland; suraj.jaiswal@lut.fi (S.J.); scott.semken@lut.fi (S.S.); aki.mikkola@lut.fi (A.M.)

**Keywords:** biomechanics, sports engineering, performance measurement, IMU sensors, XSENS suit

## Abstract

Medieval combat sport is a form of mixed martial art in which combatants engage in fighting using offensive and defensive equipment while dressed in full armor. The sport is considered extremely taxing, making it nearly impossible to maintain the same level of performance. However, this form of sport has not been thoroughly analyzed, and its impact on human physical response is largely unknown. To address this gap, the study reported here aimed to introduce and test a procedure for analyzing human physical responses within the framework of the sport. To accomplish this, two experienced combatants were asked to engage in a series of strikes, performed in the form of a set duel simulating a professional fight competition. The kinematic aspect of the procedure was examined using motion analysis with the help of an IMU suit, while the physiological aspect was evaluated based on blood lactate levels and heart rate measurements. Furthermore, an ergometer test conducted in a laboratory setting aimed to determine the lactate threshold. The duel results showed noticeable decreases in the kinematic aspects of the strikes, such as the velocity of impact, and a dramatic rise in physiological aspects, such as heart rate and blood lactate levels. During the duel sets, the blood lactate surpassed the threshold level, and at the end, the heart rate exceeded the maximum age-related level. Practicing medieval combat sport has been shown to impose an extreme physical load on the bodies of combatants, noticeably affecting their performance levels.

## 1. Introduction

In medieval combat sport, combatants fight while dressed in full armor using offensive and defensive equipment. Founded in 1990, the sport has evolved as a component of major medieval cultural events and has recently gained significant popularity. Medieval combat sport competitions can be divided into two major types: group battles and individual battles or duels. Duels are classified based on weapon type and permitted actions, with a related branch called pro-fight that has weight classifications and fewer limitations on actions. Pro-fight battles comprise three rounds of 90 s with 45 s rest intervals. The winner is determined by the number of accepted hits completed in permitted zones. The equipment used in these competitions must match their 14th to 17th century counterparts in terms of material and manufacturing methods. As a result, the armor weighs over 20 kg. The fighters implement a variety of movements similar to combat sports such as fencing, boxing, kickboxing, and wrestling while carrying the extra weight of the armor, which makes the sport physically demanding. To the authors’ knowledge, no detailed studies have investigated the impact of practicing medieval combat sport on the physical response of the combatants.

Combat sports are characterized by high neuromuscular demands that may lead to fatigue and performance deterioration, and their intense competitive nature may result in an increased degree of fatigue for all competitors [1]. This fatigue affects performance by decreasing muscular power, speed, and coordination [2]. Fatigue can also negatively affect reaction and response times and speed, which are crucial in combat sports where the outcome of the match often depends on how fast a combatant can react to a stimulus [3,4]. Given the significance of these factors, determining how performance degrades during a fight will help combatants and coaches to improve training and construct more successful competitive tactics.

Motion analysis is an effective way to examine the effect of combat matches on combatant performance. Accuracy and motion dynamics are essential for efficient combat because the goal is to always strike precisely and with maximum velocity [5,6]. Motion analysis can investigate body displacement, develop mathematical models for physical characteristics, and devise proper methods to execute accurate strikes [5,7,8]. The assessment of blood lactate concentration emerges as a crucial determinant of anaerobic performance and metabolic demands, especially in sports characterized by high-strength components such as martial arts [9,10]. Another method to monitor the athlete’s condition and exercise intensity is heart rate analysis, as it provides an indication of the athlete’s internal load and energy consumption [11,12].

While the mentioned techniques have proven valuable in analyzing the physical performance and bodily responses of combatants, certain aspects remain unaddressed. Firstly, despite separate investigations into combatant performance utilizing these methods, their combined implementation has yet to be explored. Secondly, medieval combat sport itself, and its effects on the body, has not been thoroughly examined. Therefore, this study aims to address these gaps by incorporating motion analysis, blood lactate measurement, and heart rate monitoring to explore the physical responses of a combatant engaged in medieval combat sport. The findings from examining these performance-related factors may offer essential knowledge about their impact on athlete bodies and may suggest strategies that competitors and coaches can use to minimize negative consequences. Furthermore, the methods and outcomes reported here may be applicable to other sports as well.

The objective of this study was to develop a methodological procedure for analyzing the physical responses of participants in medieval combat sport, with a focus on understanding how the sport affects performance effectiveness. To this end, the study covered the kinematic and physiological aspects of the human body to monitor the performance level of combatants. As a case example, this study considered a professional combatant with five years of experience in medieval combat sport and a professional background in judo. The combatant participated in a set duel simulating a pro-fight. The measured physiological result of the combatant from the duel set was compared with their physiological result obtained from an ergometer test in a laboratory setting.

The main research question for this study was how the practice of medieval combat sport impacts bodily responses in terms of motion analysis, blood lactate levels, and heart rate, and what the overall implications of these responses are. Furthermore, the following three hypotheses were tested and analyzed. First, the kinematic variables regarding the quality of the execution of the strikes suffer noticeably during the pro-fight. Second, the change in kinematic variables is not the same for all strikes. Some strikes are more demanding; thus, they degrade more during the pro-fight. Third, medieval combat sport puts a great deal of internal load on the combatants, which manifests through a surge in physiological factors like blood lactate level and heart rate.This study provides an understanding of the change in performance and physiological strain caused by practicing medieval combat sport. Furthermore, the results offer insights into the differing demands of various movements within medieval combat sport, enabling the combatants to make strategies to negate the unpleasant effects.

## 2. Methods for Analyzing Human Physical Responses

The responses of the human body to physical exercise can be categorized as internal and external. Internal responses relate to physiological aspects such as heart rate and blood lactate. External responses encompass kinematic and kinetic variables. Variables related to blood lactate level, heart rate, and kinematic parameters can be investigated to analyze athlete performance [2]. The chosen methods for this paper included the study of motion analysis, blood lactate measurement, and heart rate monitoring.

### 2.1. Human Motion Analysis Practice

To study motion analysis, inertial measurement units or IMUs can wirelessly deliver calibrated raw sensor data describing angular and linear motion, location, and orientation [13,14]. IMU sensors are inexpensive, portable, fast to use, and energy-efficient [15,16]. They can be used at long range (up to 150 m), be worn under clothing, and provide sufficient accuracy, especially for acceleration values [13,14]. They can be used separately located in the exact desired locations [15,16,17] or in the form of a suit with sensors being placed in predefined locations all over the body [2,11,18]. In the suit version, global rotation information, computed in Euler angles, is determined by the sensor in the hip joint [19].

IMU sensors use Bluetooth to transmit data, which enables better freedom of movement but can result in drifting caused by the absence of direct position tracking [13,14,19]. Here, the position of the person in the global coordinate system was determined with the help of premeasurement calibration [14]. Calibration, normally the process of standing in a neutral pose and walking in a straight path both forward and backward, helps to minimize drift [20,21,22,23].

When studying motion analysis in sports, and especially combat sports, establishing repeatability can be difficult. By assisting statistical analysis to remove inaccuracies, repeated recording of the same movement can help reduce random elements [5]. Establishing repeatability is not an issue when the aim is to study a single movement, because the test can be conducted in a controlled way. There have been several studies that examined the execution of particular movements in combat sports such as karate [19], taekwondo [6], boxing [24], Silat [7], and fencing [25]. However, when simulating a match condition with multiple movements, the number of random undesired factors increases. To address this problem, designing a drill or a set of predefined movements can help to reduce the randomization of the process. Drills have been used in studies to analyze performance in other sports or activities such as basketball and physical military tasks [2,11].

While conducting experimental investigations in sports, such as case studies, the study should be designed to address several key factors. Firstly, the IMU instruments need to be calibrated, and any potential sources of drift should be eliminated. The selection of instruments should ensure that the measurement frequency adequately covers the necessary aspects of the exercise. Additionally, it is essential to ensure accurate measurement of the required variables by placing sensors in critical locations, such as joints involved in the exercise. Finally, designing a drill consisting of the target movements of the sport can enhance measurement accuracy by reducing noise and mitigating other interfering factors.

### 2.2. Blood Lactate Level Measurement Approach

To measure blood lactate levels during exercise, an invasive method carried out using blood sample analyzers is the most common approach [26]. In this method, a sample of blood is taken from a body part, and its lactate value is measured. Lactate readings of blood samples taken from different locations vary. Earlobes and fingertips are the most frequently used sample locations because they produce arterialized capillary blood [27].

To better assess the intensity of the exercise, the lactate threshold should be evaluated, and three methods are commonly used [28]. The inspection method determines the threshold by examining the connection between blood lactate and workload at the time when a systematic rise in lactate concentration begins. The 0.5 mmol/L method discovers the threshold by identifying the workload before an increase of more than 0.5 mmol/L in blood lactate concentration. Next, the log–log technique determines the threshold to be where two linear lines cross on a plot of log lactate against the log of workload and the residual sum of the squares for both lines summed together is at its lowest value. Additionally, another method proposes that the beginning of blood lactate build-up can occur at a fixed blood lactate level of 4 mmol/L. Since the lactate threshold may vary from person to person, from as low as 1.4 mmol/L to as high as 7.5 mmol/L, adopting a single constant value as the average for all participants can be deceptive [27].

Blood lactate measurement can help in sport case studies by indicating the level of physical load exerted on athletes. To implement this method effectively, blood lactate measurements should be periodized to cover the stages of the procedure, allowing for an understanding of the changes in its levels. Measurements should continue for a period at the end of the experiment to ensure successful monitoring of the buildup or buffering of blood lactate in the body. Additionally, the invasive nature of lactate measurement necessitates caution to ensure safety while maintaining the accuracy of the investigation.

### 2.3. Heart Rate Monitoring Practice

Studying heart rate factors such as **$HR_{ex}$** (the heart rate at any given time during the exercise) and **$HR_{Max}$** (the highest value for the heart rate during the exercise) can also help to evaluate the resulting data [12]. **$HR_{Max}$** can be used to assess exercise intensities when a **$VO_{2}max$** (oxygen uptake) measurement is not available [29]. The maximum age-related heart rate $\left(HR_{MAR}\right)$ is a value investigated based on the age of a person with the help of the Karvonen formula, in which $HR_{MAR}=220-\left(Age\right)$ [30]. $HR_{MAR}$ especially helps when designing the exercise for laboratory settings. Furthermore, by implementing $HR_{MAR}$ in $HR_{\%}$= ($HR_{ex}$/$HR_{MAR}$) 100, it can provide a ratio for assessing the intensity of each instant of the exercise [12].

Heart rate monitoring can serve as a convenient method in sport experimental investigations, as it provides continuous measurement and is easily accessible and utilizable in forms such as chest straps. It should be considered that while designing the experiments, calculating the $HR_{MAR}$ at the initiation of the case studies can help to control the experiment in a way that $HR_{ex}$ does not exceed $HR_{MAR}$. This helps to ensure that the experiment does not put an excessive load on the bodies of the participants.

## 3. Design of Study of the Medieval Combat Sport Combatant

The procedure of this case study was designed with regard to the methodologies presented in the previous studies that implemented motion analysis, blood lactate measurement, and heart rate monitoring [2,11].

### 3.1. Study Design

The case study on the effect of practicing medieval combat sport was carried out by simulating a pro-fight of the sport and implementing the ergometer test. The procedures of this experiment were approved by the ethical committee, and it was in accordance with the Declaration of Helsinki. Before proceeding with the experiment, the whole process was explained to the participants, and they consented to the process.

### 3.2. Description of the Participant

Two medieval combat sport combatants participated in this research. The primary criterion for selecting participants was their experience in practicing medieval combat sport, ensuring both their safety and familiarity with the activity. However, only two volunteer participants were available within reach. Consequently, it was decided to organize the experiment with one participant acting as the attacker and the other as the defender. Since this study mainly focused on monitoring the attacker, only the attacker was examined. The attacker is a 179.6 cm tall 31-year-old male with a body mass of 89.5 kg, which together with the armor was 109.5 kg. He is a professional medieval combat sport combatant with a professional background in judo. His skeletal muscle mass is 41.5 kg, and his body fat mass is 16.2 kg.

### 3.3. Experimental Procedure

Two different sets of experiments in this study were carried out on two separate days. First, the study of the kinematic and physiological aspects of medieval combat sport combatant was performed in a simulated pro-fight, and then another laboratory test was carried out to retrieve information about the physiological capabilities of the combatant from an ergometer test. The participant was given 48 h to rest between tests, and he was prohibited from doing any extreme physical activity 24 h before each.

### 3.4. Instrumentation

**Figure 1:**
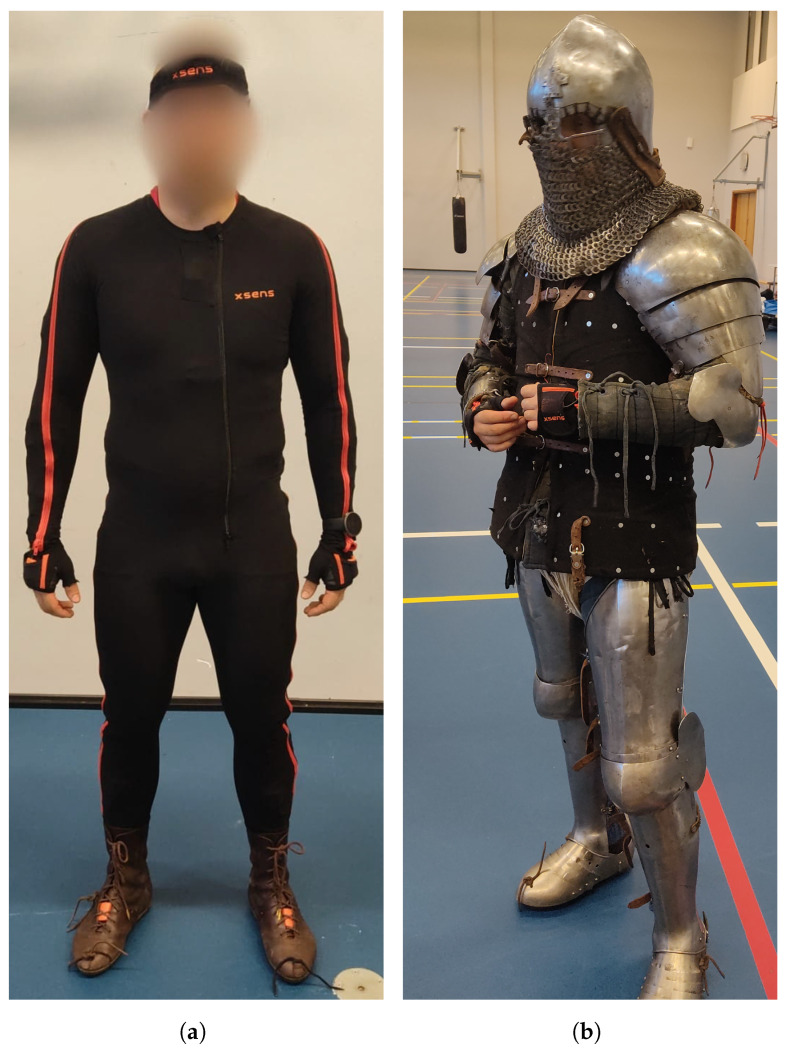
The XSENS MVN Link suit with 23 IMU sensors: (**a**) the combatant wearing the IMU suit; (**b**) the combatant in full armor on top of the IMU suit.

**Figure 2:**
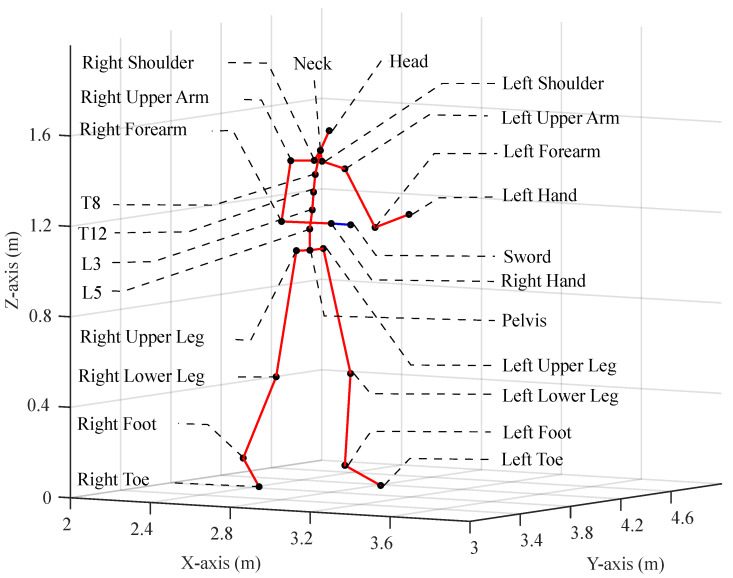
Position of different XSENS MVN Link suit sensors—23 sensors are located in different places, each representing one body part, plus one extra sensor attached to the sword. The black dots indicate the locations of the sensors, and the red lines depict the connections between them. The blue line shows the extension that was used for the sword sensor.

For the pro-fight test, the XSENS MVN Link with a sample rate of 240 Hz was used for motion analysis—see Figure 1. A suit of 23 IMU sensors worn by the participant provided information on body movements. Aside from the 23 sensors in the suit, another extra IMU sensor was attached to the sword. This sensor was connected to the right sleeve of the suit and was attached to the sword with adhesive tape. Figure 2 shows the location of the 24 sensors and the orientation of the fighter. A Firstbeat Bodyguard 2 was used to monitor heart rate, and a Senslab Lactate Scout was used to measure lactate levels.

### 3.5. Experiment Design

**Figure 3:**
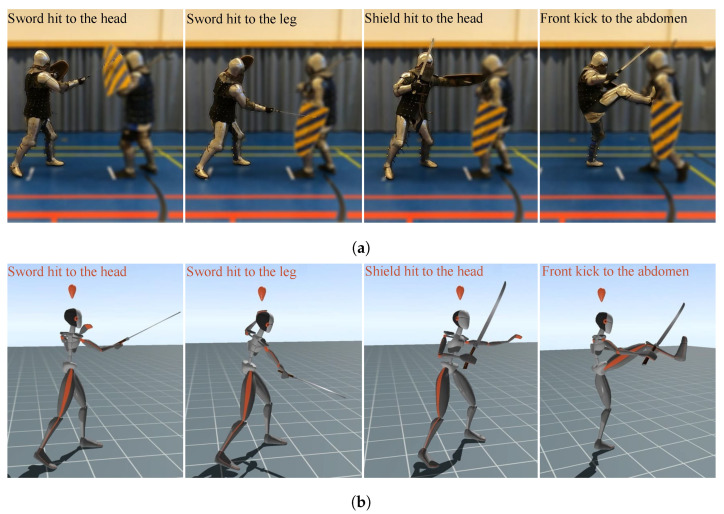
The drill for this experiment comprised four different strikes. (**a**) The execution of the drills in the real world. (**b**) The drill in the virtual world, provided by the XSENS software.

Since it was impossible to simulate a duel in a controlled way, a drill that consisted of a series of strikes common in pro-fights was designed for the pro-fight test. These were (1) a sword strike to the head, (2) a sword strike to the leg, (3) a shield strike to the head, and (4) a front kick to the abdomen—see Figure 3. The pro-fight test comprised three main phases: warm-up, maximum output (MO) rounds, and duel rounds. Both MO and duel phases consisted of three rounds. For each MO round, the combatant was asked to perform the drill once, without a time limit. In round one (R1), round two (R2), and round three (R3) of the duel, the aim was to provide a similar situation to the pro-fight, so each round lasted for 90 s. There was a 45 s rest period between each round. The participant was instructed to repeat the drill with maximum power as many times as possible.

The combatant wore the IMU suit before the warm-up. After that, the suit was calibrated before wearing the armor to avoid probable drift. Motion analysis and heart rate results were investigated continuously throughout the test. Blood lactate level was measured from the fingertips before and after the warm-up, at the end of MO rounds, and at the end of each duel round.

A bike ergometer was used for the ergometer test. The test was set up so that the workload had to increase by 25 W every three minutes. Lactate level was measured at the beginning of the test and at the end of each workload. The increase in workload continued until the combatant reached $HR_{MAR}=220-\left(Age\right)=220-31=189$ bpm. After that, the workload was dropped to 80 W, and the test continued until blood lactate levels started to decrease. As was the case for the pro-fight test, heart rate was monitored throughout the test.

### 3.6. Variables of the Study

The measured data for this research can be divided into three main categories: motion analysis, blood lactate level, and heart rate. Blood lactate and heart rate data, which were related to physiological aspects, were monitored, and their changes throughout both experiments were analyzed. For motion analysis, the kinematic variables were examined since they are essential to determine speed while carrying out various stages of movement [31]. For this purpose, the velocities of the closest sensors to the impact location were selected, and the most determining factors were chosen based on the direction of the impact. The value for those factors was calculated based on the time of impact, which was assessed by a sudden drop in velocity. Furthermore, for the selected sensors, the magnitude of the velocity vector, which represents the length or size of the vector, was calculated as the total velocity $|V_{T}|=\sqrt{V_{X}^{2}+V_{Y}^{2}+V_{Z}^{2}}$.

These velocity-related factors include the velocity of the right-hand sensor for the first strike in the Y axis ($V_{RH1Y}$) and its total velocity ($V_{RH1T}$), the velocity of the right-hand sensor for the second strike in the Y axis ($V_{RH2Y}$) and its total velocity ($V_{RH2T}$), the velocity of the left-hand sensor for the third strike in the X axis ($V_{LH3X}$) and Z axis ($V_{LH3Z}$) and its total velocity ($V_{LH3T}$), and the velocity of the right foot for the fourth strike in the X axis ($V_{RF4X}$) and Z axis ($V_{RF4Z}$) and its total velocity ($V_{RF4T}$). Furthermore, the absolute peak angular velocity of the right-upper-leg sensor for the fourth strike in the Y direction $|\omega_{Y}|$ and the location of the pelvis sensor along the Z axis $P_{Z}$ were measured.

### 3.7. Statistical Analysis of Kinematic Variables

The kinematic aspects acquired from the motion analysis needed to be analyzed and refined to be illustrated. For the MO and duel rounds, the mean values, standard deviation, and 95% confidence intervals were calculated using Microsoft Excel (version 2403), and the standard deviation was used to refine the data. The reported values for each movement for the MO rounds were the mean values for three repetitions of that movement. For R1, R2, and R3, the mean values for the average of 18 repetitions of that movement were calculated and reported as final. A confidence interval of 95% was used to show the possible variations.

## 4. Results

### 4.1. Motion Analysis

**Figure 4:**
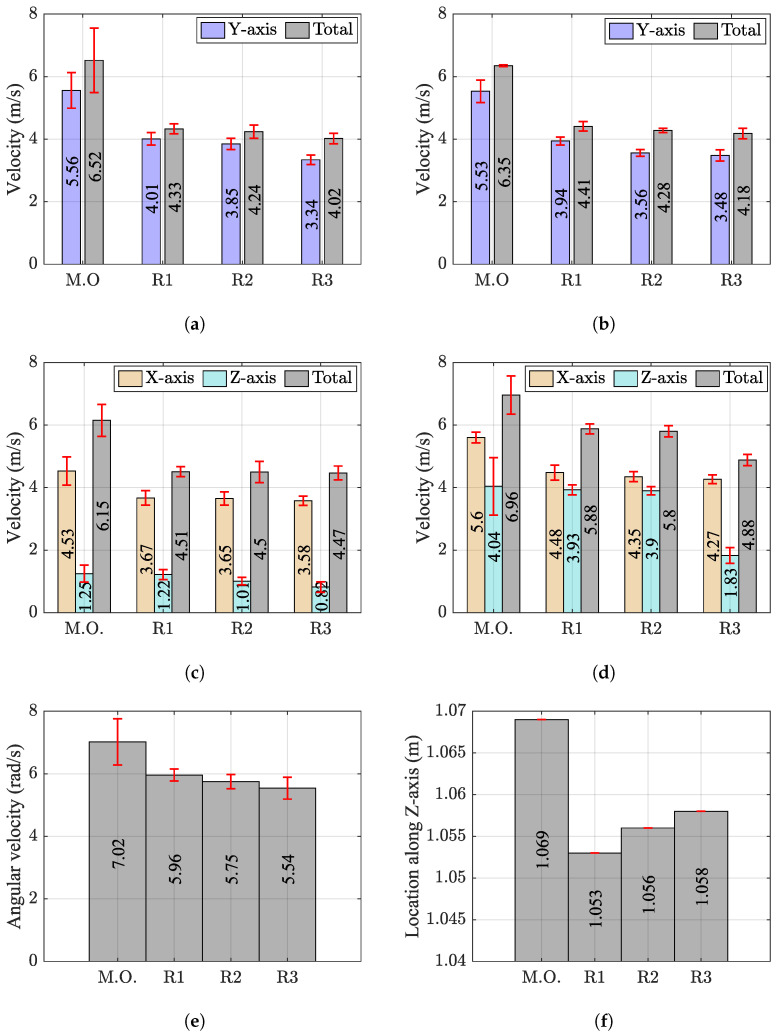
The change in the mean value of chosen kinematic variables during the pro-fight test for MO, R1, R2, and R3: (**a**) $V_{RH1Y}$ and $V_{RH1T}$ for first strike; (**b**) $V_{RH2Y}$ and $V_{RH2T}$ for the second strike; (**c**) $V_{LH3X}$, $V_{LH3Z}$, and $V_{LH3T}$ for the third strike; (**d**) $V_{RF4X}$, $V_{RF4Z}$, and $V_{RF4T}$ for the fourth strike; (**e**) $|\omega_{Y}|$ for the fourth strike; (**f**) $P_{Z}$ during the test.

Figure 4 illustrates the descriptive changes in kinematic variables during the pro-fight test. The right-hand sensor was utilized for the first and second strikes, while the left-hand sensor was chosen for the third strike due to their proximity to the impact location. Minor drifting was observed in the sword sensor after R3, likely attributed to its direct attachment to a metal object and interruptions in the magnetic sensor’s performance. Consequently, this measurement was excluded from the results. The MO demonstrated significantly higher levels of kinematic variables compared to R1, R2, and R3.

For the first three strikes, there was a consistent decrease in the mean values of $V_{RH1Y}$, $V_{RH1T}$, $V_{RH2Y}$, $V_{RH2T}$, $V_{LH3X}$, $V_{LH3Z}$, and $V_{LH3T}$ as the pro-fight test progressed. However, this decline was more pronounced for the variables of the first two strikes, which shared similarities in execution despite targeting different areas. These similarities were visible not only in the values themselves but also in the patterns of change displayed by the measured kinematic variables. In contrast, the third strike differed from the first two in terms of execution, leading to more apparent losses in $V_{LH3Z}$, although this did not significantly affect total impact velocity. See Figure 4a–c.

For the fourth strike, a noticeable decrease was observed in the mean values of $V_{RF4X}$, $V_{RF4Z}$, $V_{RF4T}$, and $|\omega_{Y}|$ from R1 to R3. Notably, there was a significant drop in the mean values of $V_{RF4Z}$ and $V_{RF4T}$ for R3. This considerable change in $V_{RF4Z}$ was particularly evident towards the end of R3, where the combatant struggled to execute the strike effectively. See Figure 4d,e.

The pelvis sensor, located at the center of the body, provides an understanding of the body’s spatial orientation. Its Z-axis location is directly linked to the location of the center of mass. Throughout the MO, the mean value of $P_{Z}$ peaked, as the combatant was not required to maintain their guard. However, during the duel, this value decreased. Subsequently, there was a slight increase during the transition from R1 to R2 and R3. See Figure 4f.

### 4.2. Blood Lactate Analysis

In the pro-fight test, the blood lactate level started at 1.0 mmol/L before the warm-up and rose to 1.6 mmol/L afterward. The time taken for calibration of the suit and wearing the armor allowed the body to engage in the buffering process. Consequently, the blood lactate value reached 1.2 mmol/L before the MO rounds and remained constant until the initiation of R1. By the end of R1, blood lactate levels surged dramatically, reaching 4.70 mmol/L. This trend persisted through R2 and R3, peaking at 6.90 and 8.20 mmol/L at the end of R2 and R3, respectively. Subsequently, the blood lactate value began to decrease, reaching 5.80 mmol/L after the initiation of the recovery phase.

**Figure 5:**
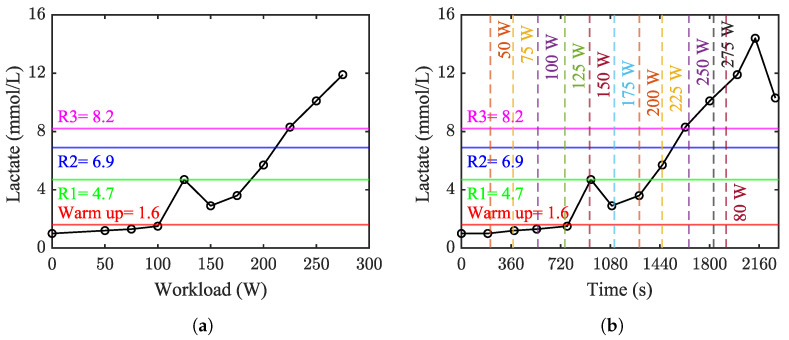
Change in the lactate level during the pro-fight and ergometer tests. The line graphs show the change in the blood lactate level for the ergometer test, while the blood lactate level of each phase of the pro-fight test is represented by horizontal lines. The vertical lines show the beginning time of each workload. (**a**) Change in lactate level against workload. (**b**) Change in lactate level against time.

Similar to the pro-fight test, the blood lactate level in the ergometer test began at 1.0 mmol/L. As the workload increased, the lactate level gradually rose, showing a steep surge at a workload of 125 W. This upward trend continued, and the peak value of 14.4 mmol/L for blood lactate was reached after decreasing the workload due to reaching $HR_{MAR}$ (see Figure 5b). At the workload of 125 W, a fluctuation occurred in the diagram, likely due to blood sample impurity, making the determination of the lactate threshold problematic (see Figure 5a). Despite this challenge, both inspection and the 0.5 mmol/L method identified the lactate threshold to be 2.9 mmol/L at a workload of 150 W.

### 4.3. Heart Rate Analysis

**Figure 6:**
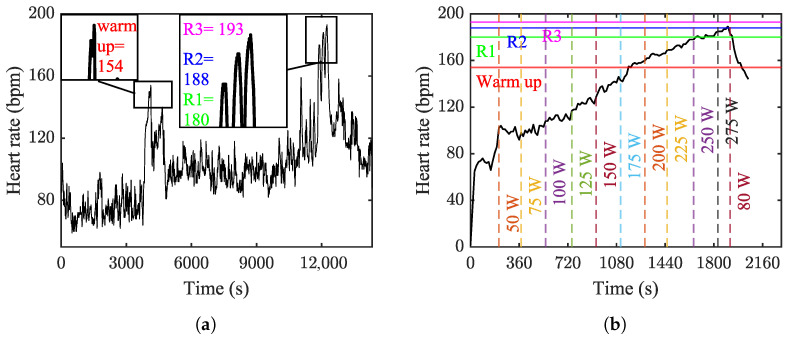
The change in heart rate value. The maximum heart rate of each phase of the pro-fight test is represented by horizontal lines, and the vertical lines show the beginning time of each workload. (**a**) The pro-fight test. (**b**) The ergometer test.

The $HR_{ex}$ results exhibit sudden increases at various points during the pro-fight test, notably during the warm-up and the duel-set stages. In the warm-up phase, $HR_{ex}$ rose, and it peaked at 154 bpm. Although it remained elevated, there was a slight decrease in $HR_{ex}$, which was followed by staying relatively constant until the initiation of the duel sets. Subsequently, $HR_{ex}$ exhibited three peaks at the end of R1, R2, and R3, reaching values of 180, 188, and 193 bpm, respectively. Throughout this period, $HR_{ex}$ consistently remained above 144 bpm, with this value representing the minimum $HR_{ex}$ observed during R2 (see Figure 6a).

In the ergometer test, the increase in $HR_{ex}$ was gradual, closely mirroring the rise in workload (see Figure 6b). This gradual increase in $HR_{ex}$ continued until $HR_{MAR}$ was reached at the workload of 275 W. At this point, with the initiation of the recovery phase, the heart rate began to decline.

## 5. Discussion and Implementation

This study aimed to introduce a procedure for analyzing human physical responses in the framework of medieval combat sport. This study’s findings confirmed the initial hypothesis regarding the degradation of the kinematic variables related to the quality of strike executions. The impact velocities for all strikes indicated signs of degradation, implying that the attacker could not maintain the same intensity as the pro-fight progressed. The decrease in the impact velocity and angular velocity of the upper leg was particularly noticeable for the fourth strike, which required extreme demands from the lower body. This loss in impact velocity is an indication of the intensity of the motion, resulting in large mechanical loads that place considerable stress on the muscular tissues [1]. The values for all kinematic variables showed a better state for the MO rounds compared to R1, R2, and R3. This was likely due to increased energy availability, the absence of time restrictions, and prolonged stance requirements.

Another measured kinematic variable was the pelvis location along the Z axis ($P_{Z}$), which gradually increased as the professional fight progressed. This rise in $P_{Z}$, which is an indication of the location of the center of mass of the combatant, suggests that the combatant was not able to maintain the same stance as the pro-fight continued. Athletes, particularly in combat sports, try to maintain a low location of center of mass to increase stability and stance. However, sustaining this stance requires consistent energy output, which becomes challenging when the combatant is already engaged in a high-intensity activity [32]. This aligns with the findings of Li et al. [2] that in basketball, fatigue leads to a rising center of mass. This effect hinders the athlete’s performance by reducing their chances of achieving a spatial and temporal advantage over their opponents. For an individual participating in a combat sport, keeping the center of mass low is a matter of greater importance, since failing to do so may result in losing balance and advantage.

The second hypothesis related to the difference in demands across distinct strike types was supported by the difference in the change in kinematic variables. The results suggested that the front kick was the most demanding, and between strikes performed by hands, the shield strike was the least demanding. One reason could be that because the right hand struck twice, it had to perform additional work in every round. Moreover, the first two strikes required more leg drive and increased trunk rotation, so they incorporated more body activity than the third strike, which mostly depended on arm extension and linear movement. This aligns with findings by Dunn et al. [33] that fatigue has a greater negative impact on boxing punches such as hooks and crosses that require more leg drive and trunk rotation compared to jabs. These less demanding movements can provide a window of opportunity for better striking, especially during fatigue.

While investigating the outputs from the IMU sensors, the drifted sword sensor rendered its acquired data inaccurate and was thus excluded from the results. Furthermore, out of the remaining 23 IMU sensors in this study, only 5 were used for investigation. When utilizing motion analysis, analyzing measurement requirements before installing sensors and proceeding with experimental studies is crucial. This ensures that sensors are placed only in necessary locations to measure required factors. Additionally, such analysis helps minimize inaccuracies, such as sensor drifting, which could jeopardize study results.

The third hypothesis was substantiated by both blood lactate and heart rate investigations. Notable increases in blood lactate and heart rate levels manifested progressively throughout the pro-fight, culminating in peak values at the pro-fight’s conclusion.

To provide an understanding of the change in the blood lactate level from the pro-fight results, the data from the ergometer test were utilized to determine the blood lactate threshold. However, it is crucial to consider that the variation in blood lactate values is highly dependent on the exercise mechanism, and it differs significantly between these two cases. The fluctuation that occurred during the ergometer test at a workload of 125 W made it challenging to identify the lactate threshold. However, the determined value of 2.9 mmol/L occurred at a workload of 150 W when the heart rate exceeded 120 bpm. This value aligns with Billat’s [34] suggestion that during the bicycle ergometer test, a rise in blood lactate level would first be noticed at heart rates of more than 120 bpm (see Figure 6b). The maximum blood lactate level during the ergometer test occurred 229 s after entering the recovery state and decreasing the workload to 80 W. This is in accordance with findings by Goodwin et al. [27], who noted that it usually takes three to eight minutes after exercise for blood lactate to reach its maximum value.

A dramatic increase in blood lactate levels occurred during the pro-fight test, where values suddenly rose above by 3.1 mmol/L and continued to increase. The increase was more significant during the transition from the warm-up and MO rounds to R1. This pattern can be explained by the increased demand for anaerobic metabolism during high-intensity activities in combat sports, which is manifested by the rise in blood lactate levels at the end of the competitions [35]. Chien et al. [26] proposed that blood lactate levels vary from 4 to 10 mmol/L for hyperlactatemia states to excessive exercise under normal conditions for the human body. The results for lactate values in R1, R2, and R3 are in complete agreement with this suggestion. Excessive lactate removal was observed at the end of both tests, which, according to Penumalla et al. [10], is caused by the body’s buffer systems. Improving the removal rate is possible through training and implementing active recovery by performing mild aerobic exercise [34,36].

The results of the heart rate monitoring indicated an extreme cardiac demand during R1, R2, and R3. According to Penumalla et al. [10], this extreme increase in cardiac output results from the blood being forced into the veins during exercise, and Andersen et al. [37] suggests that it represents the physical demands of the activity. The $HR_{Max}$ of 193 bpm, achieved at the end of R3, surpassed even $HR_{MAR}$. Maintaining such a high heart rate is almost impossible, leading to an extreme drop in the combatant’s performance. Andersen et al. [37] suggested that an average $HR_{\%}$ of 85% demonstrates the sport’s high physical demands. In this study, the mean $HR_{ex}$ during rounds R1, R2, and R3 was 170.3 bpm, and the $HR_{\%}$ associated with this value was 90.1, demonstrating the sport’s high demand for cardiac output.

Overall, the results of this study demonstrate signs of fatigue, providing a clear indication of the significant physical demands that this sport requires. Carrying extra weight due to the armor contributes to the physical demands of medieval combat sport, resulting in altered kinematic parameters, increased heart rates, and metabolic power, as observed in studies by Kessels et al. [11]. Bourdon et al. [38] suggests that this increase in metabolic power leads to a proportional increase in internal load, causing decreased cerebral blood flow in specific brain regions responsible for manual response to peripheral visual signals [4]. The effect may be more prominent in medieval combat sport due to the combatant’s limited field of view caused by the helmet.

Furthermore, fatigue may result in ACL injury, which is common among medieval combat sport practitioners, and can lead to the temporary or permanent cessation of sport activities. According to Ardern et al. [39], approximately 33% of patients with ACL injuries do not recover their pre-injury level of function, and only 55% can return to competitive sports. The resulting fatigue leads to longer muscle response periods, potentially compromising the protective function these muscles provide. Since this results in increased knee internal rotation, adduction, abduction, and rotation moments, fatigue may lead to a higher risk of noncontact ACL injuries [40].

### Limitation and Future Work

Several limitations exist in the scope of this study. First, the size and variety of the participant cohort are important factors to be considered. While the focus of this study was on highly experienced combatants, a more diverse and expansive combatant group would increase the applicability and robustness of the statistical analysis. Second, although this study concentrated on four different strikes within the realm of medieval combat sport, it should be noted that the diversity of movements surpasses these four. Execution of these strikes in a precisely identical way presents a challenge for achieving repeatability. Third, using optical motion capture compared to the IMU suit may offer better accuracy and less risk of drifting. In this case, rapid physical contact, marker detachment risk, and marker occlusion made the implementation of an IMU suit more convenient.

In future works, several modifications could be implemented to increase the depth and accuracy of the study. These modifications may include improving the depth of the study by increasing the sample size and diversity, as well as analyzing the defender. To increase the accuracy of measurements, optical motion capture could be implemented alongside the IMU sensors. Utilizing at least 6–10 cameras from selective motion capture systems such as Vicon would help mitigate marker occlusion issues. Additionally, examining ground reaction force and body temperature would contribute to a comprehensive understanding of the sport.

## 6. Conclusions

The results obtained from the pro-fight demonstrated that medieval combat sport imposes significant aerobic and anaerobic demands on the combatants, which results in fatigue and a consequent decline in performance. Motion analysis results displayed a drop in impact velocity for all strikes and a loss of proper stance maintenance during the pro-fight; while the performance deterioration varied among strikes, it may be advantageous to prioritize less affected strikes during periods of fatigue. The pro-fight resulted in a substantial increase in blood lactate levels exceeding the threshold and heart rates surpassing the age-related maximum. These factors may become even more demanding in a real pro-fight, which involves constant decision making, defensive maneuvers, and physically demanding techniques such as grappling. The fatigue induced by this sport can adversely impact performance and increase the risk of injury.

## Data Availability

The authors declare that there is no availability of data due to privacy of the participants and ethical restrictions.
